# Refinement of the Antarctic fur seal (*Arctocephalus gazella*) reference genome increases continuity and completeness

**DOI:** 10.1093/g3journal/jkae179

**Published:** 2024-07-31

**Authors:** Kosmas Hench, David L J Vendrami, Jaume Forcada, Joseph I Hoffman

**Affiliations:** Department of Evolutionary Population Genetics, Faculty of Biology, Bielefeld University, Bielefeld 33501, Germany; Department of Animal Behaviour, Faculty of Biology, Bielefeld University, Bielefeld 33501, Germany; Museum für Naturkunde - Leibniz Institute for Evolution and Biodiversity Science, Invalidenstr. 43, Berlin 10115, Germany; Department of Evolutionary Population Genetics, Faculty of Biology, Bielefeld University, Bielefeld 33501, Germany; Department of Animal Behaviour, Faculty of Biology, Bielefeld University, Bielefeld 33501, Germany; British Antarctic Survey, UKRI-NERC, High Cross, Madingley Road, Cambridge CB3 0ET, UK; Department of Evolutionary Population Genetics, Faculty of Biology, Bielefeld University, Bielefeld 33501, Germany; Department of Animal Behaviour, Faculty of Biology, Bielefeld University, Bielefeld 33501, Germany; British Antarctic Survey, UKRI-NERC, High Cross, Madingley Road, Cambridge CB3 0ET, UK; Center for Biotechnology (CeBiTec), Faculty of Biology, Bielefeld University, Bielefeld 33615, Germany; Joint Institute for Individualisation in a Changing Environment (JICE), Bielefeld University and University of Münster, Bielefeld 33501, Germany

**Keywords:** genome assembly, Antarctic fur seal, *Arctocephalus gazella*, pinniped, dovetail, whole genome alignment, Genomic Evolutionary Rate Profiling (GERP)

## Abstract

The Antarctic fur seal (*Arctocephalus gazella*) is an important top predator and indicator of the health of the Southern Ocean ecosystem. Although abundant, this species narrowly escaped extinction due to historical sealing and is currently declining as a consequence of climate change. Genomic tools are essential for understanding these anthropogenic impacts and for predicting long-term viability. However, the current reference genome (“arcGaz3”) shows considerable room for improvement in terms of both completeness and contiguity. We therefore combined PacBio sequencing, haplotype-aware HiRise assembly, and scaffolding based on Hi-C information to generate a refined assembly of the Antarctic fur seal reference genome (“arcGaz4_h1”). The new assembly is 2.53 Gb long, has a scaffold N50 of 55.6 Mb and includes 18 chromosome-sized scaffolds, which correspond to the 18 chromosomes expected in otariids. Genome completeness is greatly improved, with 23,408 annotated genes and a Benchmarking Universal Single-Copy Orthologs score raised from 84.7% to 95.2%. We furthermore included the new genome in a reference-free alignment of the genomes of 11 pinniped species to characterize evolutionary conservation across the Pinnipedia using genome-wide Genomic Evolutionary Rate Profiling. We then implemented Gene Ontology enrichment analyses to identify biological processes associated with those genes showing the highest levels of either conservation or differentiation between the 2 major pinniped families, the Otariidae and Phocidae. We show that processes linked to neuronal development, the circulatory system, and osmoregulation are overrepresented both in conserved as well as in differentiated regions of the genome.

## Introduction

The Antarctic fur seal (*Arctocephalus gazella*) is the most abundant of the eared seals (Otariidae, Chilvers 2018) and has a circumpolar distribution throughout the subantarctic zone (Forcada and Staniland 2018). This species is a top predator and keystone species that is susceptible to environmental change and serves as an indicator of ecosystem health (Boyd and Murray 2001; Krause *et al.* 2022). Over the last 3 centuries, the Antarctic fur seal has experienced a dynamic demographic history that it shares with many other pinniped species. Starting in the late $18^{\mathrm{th}}$ century, it was the target of a global sealing industry that by the 1920s had hunted this once abundant species to commercial extinction (Bonner 1958). However, this extreme demographic reduction was followed by a spectacular recovery after the cessation of sealing (Paijmans *et al.* 2020), initially because it was no longer economically viable to hunt the seals, but later due to the species being protected by law. By the early 2000s, the global population had likely surpassed its pre-sealing size, with an estimated 3.5 million individuals at South Georgia, constituting about 98% of the global population (Forcada and Staniland 2018; Hoffman *et al.* 2022; Forcada *et al.* 2023).

More recently, this trend for population growth has reversed due to the negative impacts of a rapidly changing environment. Rising sea surface temperatures have caused the seals’ primary food source (Antarctic krill, *Euphausia superba*) to shift southward (Atkinson *et al.* 2019). This has resulted in a steady decline in food availability, which has driven parallel reductions in the numbers of breeding females and pup birth weight (Forcada and Hoffman 2014; Forcada *et al.* 2023). Changes in the population size of this species can therefore be clearly linked to both historical and ongoing anthropogenic impacts through sealing and climate change. In addition to this, recovering populations of competing predator species (Trathan 2023) and the development of krill fisheries further complicate the dynamics of the krill-based food web; however, their contributions to the decline of the Antarctic fur seal population currently remain unclear (Forcada *et al.* 2023).

Population genetic and genomic studies conducted over the past 2 decades have contributed toward an improved understanding of the mating system, population structure, demographic history, and contemporary population dynamics of Antarctic fur seals. Starting with early studies of genetic diversity and population structure based on mitochondrial DNA and restriction fragment length polymorphisms (Lento *et al.* 1997; Wynen *et al.* 2000), research later shifted towards microsatellites to investigate the mating system (Hoffman *et al.* 2003, 2007) and the relationship between heterozygosity and fitness (Hoffman *et al.* 2004; Forcada and Hoffman 2014; Litzke *et al.* 2019). With the subsequent publication of the first draft reference genome (“arcGaz1”) opening the door for genomic research (Humble *et al.* 2016), more recent studies used restriction-site associated DNA sequencing and a custom single nucleotide polymorphism (SNP) array to characterize the global population structure and demographic history of this species (Humble *et al.* 2018, 2020; Hoffman *et al.* 2022) as well as to elucidate patterns of relatedness and inbreeding (Humble *et al.* 2020). Hence, population genetic research on Antarctic fur seals has steadily progressed in line with technological advances in the field.

Recent advances in genomics have also provided the opportunity to carry out comparative genomics studies. These have been used to investigate patterns of synteny across species and to identify signals of accelerated evolution in pinnipeds. Specifically, using pairwise whole genome alignments, Peart *et al.* (2021) confirmed the overall very close chromosomal synteny within the pinniped family Otariidae, while Mohr *et al.* (2022) confirmed a close synteny within phocids. Beyond this, larger multi-species alignments have been used to describe conserved genomic regions in marine mammals (Yuan *et al.* 2021) as well as to identify rapidly evolving regions of the Weddell seal and the Walrus genomes (Noh *et al.* 2022). However, there is a consensus that the assembly quality of many first-generation reference genomes limits the scope of population genomic research. Consequently, there is currently a concerted effort in marine mammal research, particularly for whales (Cetacea), to generate and improve reference genomes to achieve assembly qualities (Morin *et al.* 2020) comparable to those of the Vertebrate Genome Project (Rhie *et al.* 2021). Besides these whole genome-based approaches, studies of orthologous genomic regions across pinnipeds have revealed elevated evolutionary rates in genes involved in blubber formation and hypoxia tolerance (Park *et al.* 2018; Yuan *et al.* 2021; Noh *et al.* 2022).

Since its initial publication (“arcGaz1.0.2”, Humble *et al.* 2016), the Antarctic fur seal reference genome has undergone 2 iterations of improvements: the scaffolding of the genome was refined in 2018 by incorporating PacBio sequencing (“arcGaz1.4”, Humble *et al.* 2018) and in 2021 based on in vivo chromosome conformation capture data (Hi-C scaffolding, “arcGaz3”, Peart *et al.* 2021). However, it became evident that the genome assembly was suboptimal in terms of both completeness and contiguity, limiting its utility for population genomic research. Here, we present the next iteration of the Antarctic fur seal reference genome (“arcGaz4_h1”), which is a *de novo* assembly of the same individual used for the previous genomes. Specifically, we used PacBio and HiRise, in combination with long-range information based on Hi-C, to produce a haplotype-resolved reference genome, which has greatly improved contiguity and completeness compared to the previous versions. We believe this new assembly provides a solid basis for modern population genomic research that requires a high-quality reference genome.

Furthermore, we demonstrate the wider utility of this reference genome for pinniped research by conducting an exploratory analysis based on a reference-free multi-species whole genome alignment of 11 pinniped species, including the new Antarctic fur seal reference genome. We believe that this alignment should facilitate research on any of the included species, as well as on the group as a whole. That is because, being reference-free, the alignment can easily be expressed in the coordinates of each aligned genome and does not require any lift-over. In this study, we showcase the use of the alignment to conduct genome scans based on evolutionary conservation and differentiation between the phocids and otariids. We use these genome scans to identify evolutionary constraints shared among pinnipeds and to explore the scope for divergent evolutionary trajectories within these constrained areas.

## Materials and methods

### Tissue sampling

In order to facilitate the direct comparison of our new reference genome with previous versions, to be able to include the Hi-C structural information captured therein for additional scaffolding and ensure maximal consistency between previous and future population genomic studies of Antarctic fur seals, we opted to base the *de novo* assembly on the same individual that was already used for the initial “arcGaz1” reference genome and it’s successors (AGAZ12001, Humble *et al.* 2016). For this, we opportunistically sampled liver tissue from an adult female Antarctic fur seal that was crushed to death by a territorial bull at Freshwater Beach on Bird Island, South Georgia (54^∘^00’ S, 38^∘^02’ W) during the austral summer of 2012. Samples were transferred to RNAlater and stored at −20^∘^C for 1 month before being placed in a −80^∘^C freezer for transport back to the UK. The sample collection and export were covered by a special permit for the genome sample, issued by the Government of South Georgia and the South Sandwich Islands, Wildlife and Protected Areas Ordinance 2011 (Permit Number WPA/2013/008). It was exported from South Georgia to the UK under CITES permit 013/2012. The sample collection procedure was approved by the BAS Animal Welfare and Ethics Review Body.

### Initial genome assembly

The DNA extraction, library preparation, and initial HiRise assembly as well as the genome annotation were conducted by Dovetail genomics, as described below.

#### DNA Extraction

A total of 115 mg of skin tissue was ground and incubated in a solution of 9.5 ml G2 DNA Enhancer, RNase A and Protease for lysis. A Qiagen HMW DNA extraction kit was then used to extract at least 21.0 μg of DNA. In the extracted DNA, spooling was observed, and the DNA was dissolved in 100 μl of TE Buffer. The extracted DNA was then used to prepare PacBio circular consensus sequencing (CCS) with PacBio CCS libraries, as well as to prepare Dovetail Omni-C libraries.

#### Sequencing and *de novo* Assembly

For the initial *de novo* assembly, PacBio CCS was used to generate a total of 183.6 Gb PacBio high-fidelity (HiFi) reads. Using Hifiasm (v0.15.4-r347, Cheng *et al.* 2021) with default parameters, a phased assembly graph was created from the PacBio reads. This assembly was used to QC the Omni-C library, before deep sequencing. Hi-C integrated Hifiasm was run with default parameters using both the PacBio HiFi data and the Omni-C data.

#### Assembly Scaffolding with HiRise

To prepare the extracted DNA for the Omni-C libraries, chromatin was fixed in place in the nucleus with formaldehyde (Putnam *et al.* 2016). The chromatin was then extracted and digested with *DNase I* and chromatin ends were repaired and ligated to a biotinylated bridge adapter. Then the ends containing adapters were proximity ligated and crosslinks were reversed. Afterwards, the DNA was purified and biotin that was not internal to ligated fragments was removed. Using NEBNext Ultra enzymes and Illumina-compatible adapters, the sequencing libraries were then generated. Before PCR enrichment, biotin-containing fragments were isolated with streptavidin beads for each library. Sequencing to a target-coverage of 30X was conducted on an Illumina HiSeqX platform. The sequence reads were filtered for MQ>50 and then used for scaffolding both pseudo-haplotyes of the *de novo* assembly with HiRise (Putnam *et al.* 2016), resulting in 2 variants (one per haplotype) for the scaffolded HiRise assembly.

### Synteny-based anchoring

To make use of the large-scale structural information captured in the Hi-C-based scaffolds of the previous genome, we aligned the new haplotype assemblies onto arcGaz3. We then combined large scaffolds that unambiguously mapped onto individual arcGaz3-scaffolds into *“mega-scaffolds”*. For this, the genomes were repeat-masked using RepeatModeler (Smit and Hubley 2008) and RepeatMasker (Smit *et al.* 2013) prior to the whole-genome alignment with the last (Kiełbasa *et al.* 2011). Based on the alignment, we identified all query scaffolds within each haplotype assembly that primarily mapped to the same target scaffold in arcGaz3 and grouped them together. We only considered the 45 largest scaffolds within the haplotype assemblies for concatenation. Within those, we regarded the alignments as primary if the total alignment length on the target scaffold covered a larger share of query scaffold compared to all other possible target scaffolds and if the coverage exceeded at least 33% of the query scaffold. Primary alignments were identified and visually checked, and the coordinates were exported as bed files using a custom R script (R Core Team 2023). The identified scaffolds were then concatenated using allmaps (Tang *et al.* 2015), where grouped scaffolds were joined by 100-bp stretches of N sequence indicating an unknown gap size. The alignment-based concatenation also allowed us to identify the X chromosome within the new assemblies (Supplementary Fig. S2): based on its known identity to the California sea lion genome and the synteny with arcGaz3 (Peart *et al.* 2021), we identified and named the respective scaffold in the resulting anchored assemblies. Smaller scaffolds, as well as those that could not be unambiguously aligned, were carried over unchanged from the initial haplotype assemblies to their final anchored versions. Based on its slightly preferable scaffold N50 and BUSCO scores (evaluated based on the “carnivora_odb10” reference set, Manni *et al.* 2021), the first haplotype assembly (Anchored h1) was selected for annotation. This assembly constitutes the next iteration of the Antarctic fur seal reference genome and will subsequently be referred to as “arcGaz4_h1”, while the alternative haplotype assembly will be referred to as “arcGaz4_h2”.

### Genome annotation

#### RNA Extraction

Total RNA extraction was performed using the QIAGEN RNeasy Plus Kit following manufacturer protocols. Total RNA was quantified using Qubit RNA Assay and a TapeStation 4,200. Prior to library prep, a DNase treatment was performed, followed by AMPure bead clean up and QIAGEN FastSelect HMR rRNA depletion. Library preparation was implemented with the NEBNext Ultra II RNA Library Prep Kit following the manufacturer’s protocols. The resulting libraries were then sequenced on an Illumina NovaSeq6000 platform to create paired-end (2×150 bp) reads.

#### Repeat Masking

Repeat families in the final anchored genome assemblies were identified *de novo* and classified using the software package RepeatModeler (Smit and Hubley 2008, version 2.0.1). RepeatModeler depends on the programs RECON (Bao and Eddy 2002, version 1.08) and RepeatScout (Price *et al.* 2005, version 1.0.6) for the *de novo* identification of repeats within the genome. The custom repeat library obtained from RepeatModeler was used to discover, identify, and mask the repeats in the assembly file using RepeatMasker (Version 4.1.0).

#### Gene Annotation

Coding sequences from *Canis lupus familiaris*, *Mirounga angustirostris* and *Zalophus californianus* were used to train the initial *ab initio* model for the Antarctic fur seal using the AUGUSTUS software (Stanke *et al.* 2008, version 2.5.5). Six rounds of prediction optimization were performed with the software package provided by AUGUSTUS. The same coding sequences were also used to train a separate *ab initio* model for the Antarctic fur seal using SNAP (Korf 2004, version 2006-07-28). RNAseq reads were mapped onto the genome using the STAR aligner software (Dobin *et al.* 2013, version 2.7) and intron hints were generated with the bam2hints tool within the AUGUSTUS software. MAKER (Cantarel *et al.* 2008), SNAP, and AUGUSTUS (with intron-exon boundary hints provided from the RNA-Seq data) were then used to predict genes in the repeat-masked reference genome. To help guide the prediction process, Swiss-Prot peptide sequences from the UniProt database (The UniProt Consortium 2015) were downloaded and used in conjunction with the protein sequences from *C. lupus familiaris*, *M. angustirostris*, and *Z. californianus* to generate peptide evidence in the Maker pipeline. Only genes that were predicted by both SNAP and AUGUSTUS were retained in the final gene sets. To help assess the quality of the gene prediction, annotation edit distance scores were generated for each of the predicted genes as part of the MAKER pipeline. Genes were further characterized for their putative function by performing a BLAST (Camacho *et al.* 2009) search of the peptide sequences against the UniProt database. tRNAs were predicted using the software tRNAscan-SE (Chan *et al.* 2021, version 2.05).

#### Localization of the *MHC* Class II *DQB* Exon 2 and SNP Array Loci

We sought to locate in the reference genome the *MHC* class II *DQB* exon 2 locus described by Tebbe *et al.* (2022) as well as the SNP loci present in the custom Antarctic fur seal 85K SNP array developed by Humble *et al.* (2020). To do so, we used bwa mem (Li 2013) to align the *MHC* class II *DQB* exon 2 consensus sequence and the 71 bp flanking sequences of all of the SNP loci to the 2 haplotypes of the reference genome separately. In both cases, we then retained only unique alignments with a mapping quality greater than 30. Next, we compared the *MHC* class II *DQB* exon 2 sequences present in the reference genome to the 14 alternative alleles described by Tebbe *et al.* (2022). Subsequently, we quantified the proportion of SNPs present in the array that could be localized in arcGaz4_h1 and arcGaz4_h2.

### Phylogenetic context

To provide a comparative perspective on arcGaz4_h1 and to place it into a phylogenetic context, we conducted an exploratory analysis characterizing broad patterns of genomic conservation across the Pinnipedia.

#### Whole Genome Alignments

**Fig. 1.**
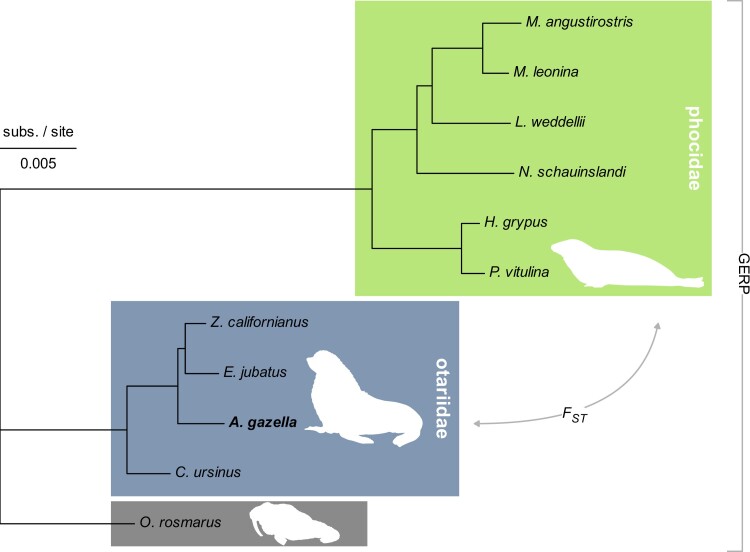
Neutral phylogeny of the subset of analyzed pinniped species. The topology of the phylogeny is based on TimeTree 5 data and is restricted to pinniped species with a reference genome in NCBI. The branch lengths are given in substitutions per site and were estimated from a whole genome alignment, using 5,000 windows with 1 kb length of non-coding sequence. The subset contains 4 genomes of the family Otariidae (eared seals, highlighted central clade), 6 genomes of the family Phocidae (earless seals, highlighted clade on top) and the walrus genome. The position of the Antarctic fur seal (*A. gazella*) is highlighted in bold. In the comparative analysis, conservation scores [Genomic Evolutionary Rate Profiling (GERP)] are based on the alignment of all 11 pinniped genomes, while genetic differentiation ($F_{ST}$) was computed between the otariids and phocids. The walrus was excluded from the $F_{ST}$ calculation due to its distinct evolutionary history being the sole extant representative of the third pinniped family Odobenidae. The pinniped art in this figure was created by Rebecca Carter (www.rebeccacarterart.co.uk) and is reproduced with her permission. All rights reserved.

We selected those pinniped species with a reference genome available in NCBI (accessed 2023-03-22) and that were also included in the dataset of TimeTree 5 (Kumar *et al.* 2022). The reference genomes were downloaded using the NCBI program datasets and aligned with the progressive-cactus pipeline (Armstrong *et al.* 2020), using the TimeTree 5 pinniped topology for guidance (Fig. 1, Supplementary Table S1). The resulting alignment was in the hierarchical alignment (hal) format (Hickey *et al.* 2013), which contains the genomic sequences of all of the species, their relationships to each other, and their underlying phylogenetic topology. To update this topology to the neutral phylogeny of the aligned species (required for the estimation of genomic conservation, see below), we created a maximum likelihood-based phylogeny informed by the genome alignment. We used a combination of the cactus command halAlignmentDepth, wig_to_bed from BEDOPS (Neph *et al.* 2012), and bedtools (Quinlan and Hall 2010) to extract 5,000 random windows with 1 kb length from the alignment. These were constrained to exclude coding sites (based on the genome annotation) and regions where more than one genome was missing from the alignment (requiring a minimum coverage of 10). The alignment was converted from hal to maf format using the cactus command cactus-hal2maf. From this, the random windows were extracted using a combination of maffilter (Dutheil *et al.* 2014) and SeqKit (Shen *et al.* 2016) to create a single concatenated multi-fasta file. This was used as input for the estimation of the branch lengths of the phylogeny with iqtree (Minh *et al.* 2020), using the TimeTree 5 topology as a constraint.

#### Genomic Conservation and Differentiation

We used gerpcol (Davydov *et al.* 2010) to conduct the GERP scoring across all 11 pinniped genomes, including the walrus. Using the maf version of the whole genome alignment as input, the evolutionary constraint in terms of rejected substitutions (RS score) was calculated for all sites of the alignment with a coverage of at least 3 genomes. To characterize genetic differentiation between the Otariidae and the Phocidae (excluding the walrus), we extracted 187,315,308 SNPs from the alignment using the cactus tool halSnps, which we further converted into vcf format using custom R and bash scripts. Genetic differentiation ($F_{ST}$) was computed using the version of vcftools (Danecek *et al.* 2011) that was modified by Dutheil (2023) to be compatible with haploid genotypes. Note, that the interpretation of $F_{ST}$ as an indicator of selection is limited, particularly in cases of correlated co-ancestry, as in the presented phylogeny (Bierne *et al.* 2013). The estimation of genetic differentiation is thus primarily intended as auxiliary information to the GERP scores and not as a thorough scan for signals of selection. Both the GERP scores and the differentiation results were then averaged within 3 sets along the genome, namely, broad sliding windows (50 kb width, 25 kb increments), fine sliding windows (10 kb width, 5 kb increments), and within the identified BUSCOs in arcGaz4_h1. The averaging was done using a combination of the bedtools commands makewindows and intersect, as well as custom R scripts. For each of these sets, the alignment coverage was summarized using halAlignmentDepth, bedtools, and R. For each window, we averaged the overall alignment coverage as well as the coverage of the genomes from each pinniped family. Furthermore, for each window/BUSCO, we quantified the percentage of the alignment exceeding a specific target coverage of 4 genomes for the combined species set and 2 genomes within each pinniped family. Subsequently, we used these summaries to restrict our outlier analysis to windows that, on average, were covered by at least 2 genomes per family for at least 50% of the window and with an SNP density exceeding 1%.

#### Gene Ontology Enrichment Analysis

Using the available gene ontology (GO) term annotation of the BUSCOs provided by OrthoDB (v10, accessed on the 2023-11-30, Ashburner *et al.* 2000; Kriventseva *et al.* 2019; The Gene Ontology Consortium *et al.* 2023), we tested for GO enrichment among highly conserved and among strongly differentiated genes. To generate the full BUSCO set for the GO term enrichment analyses, we subsetted the BUSCO results to those classified as “complete”, with a minimum average coverage of 2 genomes for both pinniped families and a minimum SNP density of 1 SNP per 100 bp. Within this subset, we selected the most conserved and most differentiated BUSCOs based on the $99^{\mathrm{th}}$ percentile of the average GERP and $F_{ST}$ scores, respectively, to create a “top-GERP” and a “top-$F_{ST}$” BUSCO set. Then, we annotated the full BUSCO set with the respective GO terms. We then used the R package topGO to conduct 2 tests to search for GO terms enriched in either the top-GERP or the top-$F_{ST}$ BUSCO set. Specifically, we used the Fisher’s exact test implementation with the elimCount algorithm and a min_node_size of 5. This means that we tested for the presence or absence of GO terms within the top BUSCO sets, taking the GO graph structure into account and truncating the GO graph to include only those GO terms that contained at least 5 BUSCOs. The enrichment test results were sorted by statistical significance and the top 10 GO terms with the lowest *p* values were reported for each test. A detailed description of top GO terms was then extracted from QuickGO (accessed on the 2023-12-13, Binns *et al.* 2009). Finally, BUSCOs linked to the top GO terms were extracted and their GERP and $F_{ST}$ profiles were compared with the full BUSCO background.

### Software versions

With the exception of the assembly procedure implemented by Dovetail and the localization of the *MHC* class II *DQB* exon 2 and SNP array loci, all of the analyses were managed using snakemake (Mölder *et al.* 2021) in conjunction with apptainer containers (Kurtzer *et al.* 2017) or conda environments (Anaconda Software Distribution 2020). For these parts of the analysis, version numbers of the used software programs are omitted for readability. However, the complete computing environments and all program settings for these analyses are documented and provided alongside the code (see Code availability statement).

## Results and discussion

### Assembly quality

Compared to the previous *A. gazella* reference genome (arcGaz3, Peart *et al.* 2021), the initial HiRise assemblies include an order of magnitude fewer scaffolds (557 & 381 vs 5,180) are slightly longer in total (2.53 & 2.52 vs 2.31 Gb) and have higher contig N50s (56 & 74 vs 0.5 Mb). Furthermore, the new assemblies have substantially improved completeness, with the number of missing BUSCOs being reduced by approximately two-thirds (95.2 and 95.5 vs 84.7%, Table 1) In general, these values indicate a substantial increase in both the continuity and completeness of the new assemblies. We attribute these improvements to a number of factors: (i) the switch to PacBio sequencing, which provides longer reads for the initial assembly; (ii) the use of haplotype-aware assembly methods, which reduce the ambiguity caused by heterozygosity in the reference individual, and (iii) the use of HiRise technology for intermediate-scale scaffolding.

**Table 1.** Quality metrics for the new HiRise assemblies

Genome	Total size (bp)	n Scaffolds	Contig N50 (bp)	Scaffold N50 (bp)	Complete BUSCOs
arcGaz3	2,300,877,616	5,180	477,984	139,181,869	84.7%
HiRise_h1	2,527,997,584	580	55,559,406	83,418,100	95.2%
HiRise_h2	2,517,684,524	406	73,963,075	83,478,833	95.0%
arcGaz4_h1	2,527,999,884	557	55,559,406	141,635,559	95.2%
arcGaz4_h2	2,517,687,024	381	73,963,075	141,085,310	95.0%

The previous *A. gazella* assembly (arcGaz3) is included for context. Complete BUSCOs refer to the combined percentage of complete single-copy and duplicated BUSCOs of the “Carnivora” reference set including a total of 14,502 BUSCOs.

However, while the vast majority of arcGaz3 consists of 18 large scaffolds, corresponding to the 18 chromosomes expected within otariid genomes (Beklemisheva *et al.* 2020), 35–40 scaffolds of the new genome are necessary to compile a comparable share of the assemblies (Supplementary Fig. S1). We reasoned that the new assemblies were likely split at long repetitive regions, such as the centromeres, which were spanned by the previous genome. In fact, arcGaz3 owes it is impressive scaffold N50 to Hi-C-based scaffolding, which substantially increased the N50 compared to its predecessor from 6.2 Mb (Humble *et al.* 2018) to 139.2 Mb (Peart *et al.* 2021). As the previous reference genome was based on the same individual (SAMN04159679), we therefore used synteny-based anchoring to recapture the structural information provided by Hi-C and to improve the overall continuity of the final assembly. This resulted in the scaffold N50s of the anchored haplotype assemblies slightly surpassing that of arcGaz3 (141.6 & 141.1 vs. 139.2 Mb).

**Fig. 2.**
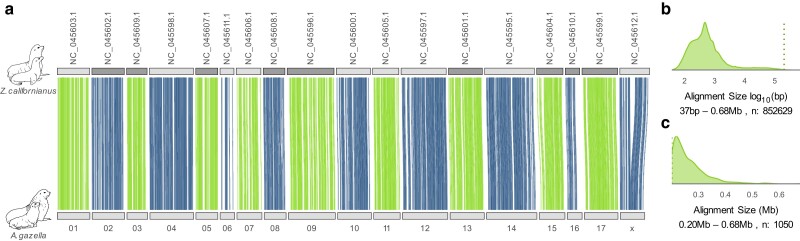
Broad scale synteny between the Antarctic fur seal (arcGaz4_h1) and the California sea lion reference genome. a) The whole genome alignment of the Antarctic fur seal (*A. gazella*, bottom, “01”–“x” refers to scaffolds mscaf_a1_01–mscaf_a1_x) and the California sea lion (*Z. californianus*, top). The gray bars indicate the 18 major scaffolds of the respective genomes and the blue and green lines indicate sequence alignments larger than 0.2 Mb. Dark gray bars in the California sea lion genome indicate chromosome alignments that were reversed to facilitate the visual representation. b) Size distribution of the sequence alignments for the full set of alignments on a log scale. The dotted line in indicates the 0.2 Mb threshold. c) Size distribution of the alignment subset larger than 0.2 Mb on a linear scale. The pinniped art in this figure was created by Rebecca Carter (www.rebeccacarterart.co.uk) and is reproduced with her permission. All rights reserved.

In a direct comparison, the 2 initial HiRise haplotype assemblies are very similar in terms of assembly size (both 2.5 Gb), the number of scaffolds (580 & 406) and completeness (Table 1). Anchoring based on the same reference further increased structural similarities between the 2 haplotypes and streamlined the arrangement of the scaffolds within the assemblies (Table 1, Supplementary Fig. S2). Furthermore, in both of the haplotype assemblies, the vast majority of the sequence is contained within the largest 18 scaffolds (94.3 & 93.8%, Supplementary Fig. S2). Close synteny between the California sea lion and the Antarctic fur seal was already known based on arcGaz3 (Peart *et al.* 2021), and accordingly this close match carried over to arcGaz4_h1 (Fig. 2). As the identities of the chromosomes in the California sea lion genome have already been unequivocally established using chromosome painting (Peart *et al.* 2021), we regard the 18 mega-scaffolds of arcGaz4_h1 and arcGaz4_h2 as representations of the 18 chromosomes expected for otariids (Beklemisheva *et al.* 2020). Finally, these scaffolds also carry the vast majority of the complete BUSCO groups (98.3 & 96.8%).

We note that the definition of haplotype 1 and haplotype 2 is merely a technical way of separating each of the 2 chromosomal haplotypes present in the diploid genome of the reference individual. The complete set of chromosomal haplotypes captured within the 2 assemblies does not hold any biological meaning, as the sorting of a particular chromosomal haplotype into assembly set 1 or 2 happened arbitrarily and because there is no linkage across chromosomes. Consequently, there are no meaningful connections among individual scaffolds within each haplotype assembly. For example, the first scaffold of arcGaz4_h1 (mscaf_h1_01) is not more strongly associated with mscaf_h1_02 than it is with mscaf_h2_02.

To summarize, arcGaz4 represents an improved version of the reference genome of the Antarctic fur seal compared to its predecessors. Both haplotype assemblies are essentially equivalent in terms of assembly quality and content. Haplotype 1 was therefore selected as the reference genome because it was slightly superior, mainly in terms of completeness. In cases where concerns about reference-bias for the chosen haplotype exist, the 2 haplotypes could be combined into a miniature pan-genome, using minigraph-cactus (Hickey *et al.* 2023). However, we refrain from doing so at the current time, based on our judgment that a true pangenome would require the inclusion of more than 2 haplotypes.

### Assembly content

The annotation of arcGaz4_h1 identified a total of 23,408 gene predictions with an average length of 1.37 kb, spanning a total of 32.1 Mb (1.27% of the assembly). Of the predicted genes, 94.2% reside within the largest 18 scaffolds. Beyond these gene predictions, we also identified a set of loci that have been the focus of previous studies of *A. gazella*. The bwa alignment of the *MHC* class II *DQB* exon 2 consensus sequence allowed us to uniquely identify the location of this exon within the genome. Specifically, the *MHC* class II *DQB* exon 2 is located on the $13^{\mathrm{th}}$ mega-scaffold of both haplotype assemblies (mscaf_a1_13 29,778,656–29,778,924 and mscaf_a2_13 29,829,913–29,830,181). This is in accordance with the genome annotation, which places the gene model for *HLA-DQB1* on mscaf_a1_13 (bp 29,775,965–29,781,590). By comparing the sequence of the *MHC* class II *DQB* exon 2 present in the 2 haplotypes of the reference genome to the alternative alleles described by Tebbe *et al.* (2022), we could show that the individual used to produce the reference genome is heterozygous at this locus. Specifically, it carries one copy of allele 4 (*ArGa-DQB-4*) and a new allele that was not present in the pool of individuals analyzed by Tebbe *et al.* (2022). This haplotype shows the greatest similarity to haplotype 5 (*ArGa-DQB-5*), differing by 5 bases.

The alignment of the flanking sequences of the SNPs present on the 85K SNP array showed that the vast majority of them could be located in the new reference genome. Specifically, only 143 SNPs (0.17%) and 230 SNPs (0.27%) could not be mapped to arcGaz4_h1 and arcGaz4_h2, respectively. Moreover, when combining the mappings to the 2 haplotypes, only 66 SNPs (0.08%) could not be located in the new reference genome. Hence, genomic location information could be retrieved for more than 99% of the SNPs present on the Antarctic fur seal SNP array.

### Phylogentic context

To demonstrate the new Antarctic fur seal reference genome’s potential for comparative population genomic studies of pinnipeds, we conducted an exploratory analysis of genomic conservation patterns among pinnipeds. The aim was both to further describe the new genome within its “evolutionary neighborhood” and to showcase its utility for generating and testing hypotheses in a comparative context. Of particular interest for pinniped evolution are constraints and differences in traits linked to the main physiological challenges that pinnipeds as a group had to adapt to when transitioning from a terrestrial to a marine lifestyle. These include apnea and diving physiology, sensory physiology, osmo- and thermoregulation, fasting, and lactation physiology (Crocker and Champagne 2018).

**Fig. 3.**
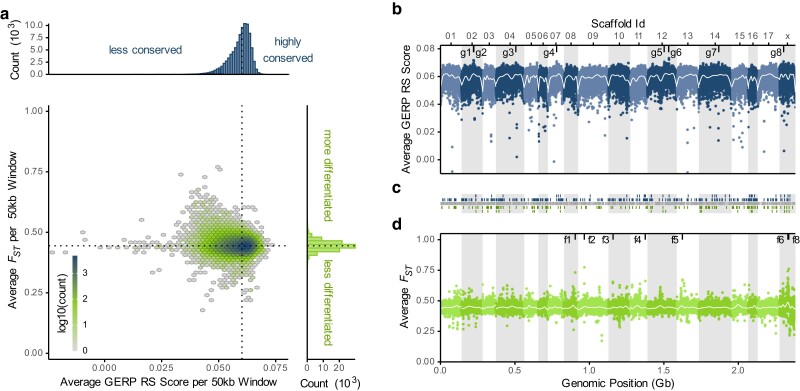
Sliding window summary of GERP scores in pinnipeds and $F_{\mathrm{ST}}$ between otariids and phocids. a) Bivariate and marginal histograms of the GERP scores (*RS*, *x*-axis/top) and $F_{ST}$ values (*y*-axis/right). The color of the bivariate histogram indicates the $log_{10}$ of the window count for the respective bin. The dotted lines indicate the median values for each axis. b) Sliding window summary of GERP scores along the genome. Alternating colors and backgrounds indicate the 18 major scaffolds with the scaffold labels on top (“01”–“x” refers to scaffolds mscaf_a1_01–mscaf_a1_x). The white line indicates a GAM-based smoothing of the GERP values. Inward tick marks and their labels indicate outlier peaks located above the $99.99^{\mathrm{th}}$ percentile of the distribution of values. c) Genomic locations of the BUSCOs classified as complete. The central gray track displays the full BUSCO set. The blue tracks on the top indicate those BUSCOs identified by the GERP-based GO term enrichment analysis. Specifically, the upper blue track shows the 1% of BUSCOs with the highest GERP values, while the lower blue track shows all BUSCOs with links to the GO terms identified based on the upper track. Similarly, the bottom green tracks are BUSCOs identified by the $F_{ST}$ based GO term analysis, with the bottom track containing the 1% most extreme BUSCOs and the central track all BUSCOs with links to the identified GO terms. d) Sliding window summary of $F_{ST}$ values along the genome. Alternating colors and backgrounds indicate the 18 major scaffolds, with the bottom tick marks indicating their base pair positions on the concatenated genome in gigabases (Gb). The white line indicates a GAM-based smoothing of the $F_{ST}$ values. Inward tick marks and their labels indicate outlier peaks located above the $99.99^{\mathrm{th}}$ percentile of the distribution of values. Conservation (GERP) and differentiation ($F_{ST}$) scores were averaged within 50 kb windows with 25 kb increments. Windows with an alignment coverage below 2 genomes (within either seal family) or a SNP density below 1 SNP / 100 bp were omitted.

Using cactus, we successfully aligned all 11 pinniped genomes for 79.2% of the Antarctic fur seal genome, and only 1.5% of the genome did not align with any other genome. Unsurprisingly, the other otariid genomes aligned better compared to the more distant phocid genomes (95.0 vs 88.8% of the genome with an alignment depth of 4, Supplementary Fig. S3). This whole genome alignment served as the backbone for all of the following analyses, including the estimation of branch lengths in the pinniped phylogeny. Using a concatenation of 5,000 windows with 1 kb length of non-coding sequence, we inferred branch lengths for putative neutrally evolving nuclear sequences within our subset of the pinniped phylogeny (Fig. 1). The cumulative branch length of this neutral phylogeny (0.086) represents the expected rate of substitutions per site within the GERP conservation scoring, where it defines the upper bound of possible RS scores in our study (Cooper *et al.* 2005). The observed median throughout the 50 kb windows along the genome was an RS score of 0.060, with the distribution being slightly skewed towards lower RS scores and 68% of the windows having RS scores between 0.054 and 0.063 (corresponding to the 2σ interval around the median, Fig. 3a).

Focusing on conservation scores along the genome, GERP scores appear to be reduced towards the edges of the large scaffolds (Fig. 3b). A large-scale structural effect of the position on the chromosome seems plausible, given that distance from the centromere affects both mutation (Chen *et al.* 2010) and recombination rates (Peñalba and Wolf 2020; Stevison and McGaugh 2020) and thus directly impacts the speed at which a sequence can evolve and diverge. Given that pinniped karyotypes are generally characterized by meta- and acrocentric chromosomes, we can assume that the centromeres lie in the more central regions of the large scaffolds and that peripheral regions are more distant from the centromeres (Beklemisheva *et al.* 2020). Another influencing factor might be large-scale variation in the alignment coverage, which shows parallel drops in some of the scaffold edges (e.g. mscaf_a1_03 and mscaf_a1_06, Supplementary Fig. S4). However, most of the terminal drops in the conservation scores seem not to be influenced by alignment coverage.

While for most of the genome, conservation scores remained well below an average RS score of 0.066, we identified a few peaks exceeding background levels and reaching average RS scores up to 0.073. A scan of the windows with the 0.01% most extreme GERP values showed that half of these GERP outlier regions (g1, g3, g5, and g6, Fig. 3b) did not include any gene model within the area of elevated GERP scores (Supplementary Fig. S5). The other peaks corresponded to genomic regions containing the genes *OTX1* (g2), *SOX2* (g7), *THOC2* (g8), and the *HOXA*-cluster (g4). In humans, all of these genes have been linked to important developmental processes, with *OTX1* being important for the development of the brain and sense organs, *SOX2* controlling the expression of genes involved in embryonic development, *THOC2* being involved in neuronal development and the *HOXA*-cluster playing a major role in the developmental organization of the anterior-posterior axis (The UniProt Consortium 2023). It seems plausible that the involvement of these genes in the regulation of core developmental processes might restrict evolutionary variability within pinnipeds. In fact, this conservation likely extends further into mammals and vertebrates more generally.

In terms of differentiation between the otariid and phocid genomes, the genome-wide median average windowed $F_{ST}$ is 0.44, with 68% of the windows falling between 0.43 and 0.46 (Fig. 3a). Throughout the genome, differentiation appears quite homogenous, although a handful of peaks with average $F_{ST}$ values of around 0.6 stand out. However, due to the small number of haplotypes within each group, the $F_{ST}$ analysis is susceptible to the effects of uneven coverage within the alignment and should therefore be interpreted with caution (see also Supplementary Fig. S6). Nonetheless, after filtering for minimum coverage (see [sec:materials:methods]Methods), we investigated the most extreme $F_{ST}$ peaks that exceeded the $99.99^{\mathrm{th}}$ percentile of $F_{ST}$ values within windows. This identified 8 outlier peaks (f1–f8, Fig. 3d), 5 of which did not contain any gene models (f2 and f5–f8, Supplementary Figure S6). The remaining windows contained models for a set of Glutathione S-transferases (“GSTs”, f1: *GSTT4*, *GSTT1*, and *GSTT2B*) and for *ADAM20* (f3) and *OR4C6* (f4).

GSTs play a key role in detoxification processes under oxidative stress, while *ADAM20* in humans is linked to sperm maturation and fertilization and *OR4C6* represents an odorant receptor (The UniProt Consortium 2015), so differentiation between the pinniped families is plausible (O’Rand 1988; Dobson and Jouventin 2003; Stoffel *et al.* 2015; Carlisle and Swanson 2021). Causes for differentiation may be least obvious for the GSTs, as efficient detoxification appears to be a generally beneficial trait. Yet, while there is substantial variation within both families, otariids and phocids famously differ in their diving capabilities, with phocids generally being capable of much longer and deeper dives compared to otariids (Berta 2018). Prolonged dives imply increasing oxygen limitation in the pinniped brain (Clanton and Klawitter 2001; Larson *et al.* 2014), and indeed, signatures of positive selection on hypoxia signaling genes have been reported in otariids and phocids, as well as in the walrus (Foote *et al.* 2015; Park *et al.* 2018; Noh *et al.* 2022). Yet due to their more extreme diving behavior, this challenge is expected to be more severe in phocids.

To explore biological processes that are either conserved or divergent across the pinnipeds, we conducted 2 GO term analyses. These enrichment tests were based on BUSCOs that exceeded the $99^{\mathrm{th}}$ percentile of either the GERP score or $F_{ST}$ (Fig. 3c) distributions. For each test, we selected the top 10 GO terms with the most extreme *p* values of the enrichment test for further characterization. Many of the GO terms identified in the enrichment analysis are involved in neuron and brain development, and in the circulatory system, with links to both oxygen supply and osmoregulation (Supplementary Table S2 and Table S3).

**Fig. 4.**
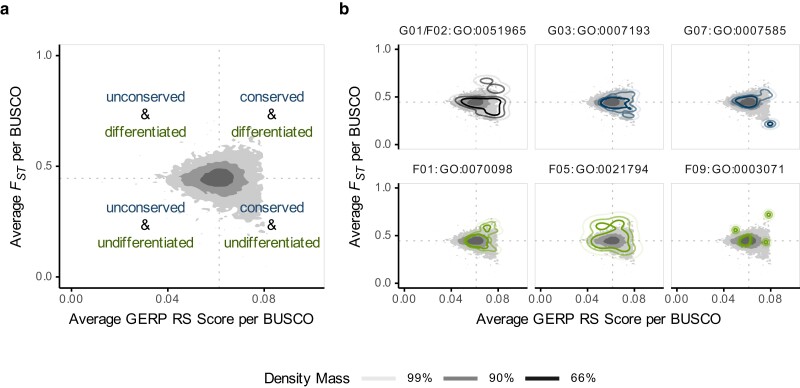
Bivariate density distribution of average GERP scores and $F_{\mathrm{ST}}$ among BUSCOs. The bivariate densities summarize the average conservation scores (*x*) and $F_{ST}$ values (*y*) among BUSCOs. The dotted lines indicate the median GERP and $F_{ST}$ values of the full BUSCO set. a) Density distribution of the full BUSCO set, with the shading indicating the share of the density mass covered. b) Density distributions for BUSCOs with links to top GO terms identified by the enrichment analyses. For context, the density of the full set from panel a) is given in the background. Blue density lines indicate GO terms identified by the GERP-based enrichment analysis, while green lines indicate those identified by the $F_{ST}$ based analysis. The GO term GO:0070098 is indicated in black, as it is included in both GO term sets. The full set of identified GO terms is shown in Supplementary Fig. S7.

Strikingly, the most significant GO term in the GERP-based enrichment analysis (GO:0051965, G01) was also the second most significantly enriched term in the $F_{ST}$-based analysis (F02). Enrichment both within conserved and differentiated BUSCOs might initially appear paradoxical (Fig. 4, Supplementary Fig. S7). However, it is not for 2 reasons. First, multiple BUSCOs are connected to the same GO term. A specific GO term can therefore contain a set of BUSCOs that can include both conserved and differentiated BUSCOs. Second, the GERP scores were compiled for the entire sequence alignment, while $F_{ST}$ values were only computed for variable sites. A single BUSCO (or any genomic window) can therefore be both highly conserved and strongly differentiated if it contains comparably few SNPs, so long as the variation in the SNPs is distinctly partitioned between the 2 seal families.

The bivariate distribution of GERP and $F_{ST}$ values for BUSCOs involved in GO:0051965 show that they are indeed characterized by comparably high conservation scores. Despite showing up as the second most significant term in the $F_{ST}$-based test, most of the involved BUSCOs seem to be comparably uniform within the pinniped clade, with rather average and even low $F_{ST}$ values. Yet, a subgroup of BUSCOs forms a distinct cluster that leads to an over-representation of this GO term within the set of more differentiated BUSCOs (Fig. 4b). Our interpretation is that BUSCO genes involved in synapse assembly are subjected to strong evolutionary constraints in the pinnipeds. Nonetheless, discrete feasible alternatives may exist for some of the involved genes, and whether due to chance or adaptation, the 2 pinniped families apparently carry different alleles for them. Similar patterns occur for other GO terms with links to neuronal or brain development (GO:0007193 (G03), GO:0051386 G09, Fig. 4b), while GO:0021794 (F05) was only enriched in the differentiation-based analysis and includes many BUSCOs with more relaxed conservation scores (Fig. 4b). Despite the generally expected strong conservation on mammal brain development, the transition from a terrestrial to an aquatic lifestyle imposed new constraints on the pinnipeds, including the necessity to adapt to frequent periods of hypoxia induced by their diving behavior (Schneuer *et al.* 2012; Larson *et al.* 2014). The observed pattern suggests that pinnipeds adapted within the narrow scope imposed by the evolutionary constraints. Indeed, it points to candidate genes where, despite the strong constraints, otariids and phocids may have realized different solutions.

The second reoccurring theme that was picked up by both GO term enrichment analyses was a connection to the circulatory system and includes the GO terms GO:0007193 (G03), GO:0007585 (G07), GO:1902075 (G10), GO:0001991 (F03) and GO:0003071 (F09, Supplementary Table S2 and Table S3). The cAMP-mediated signaling pathways linked to GO:0007193 among other effects also mediate water uptake in the gut and kidney, which influences osmoregulation (Xiaodong *et al.* 2008). All other osmoregulation-linked GO terms are characterized by relatively weak differentiation between the otariids and phocids (Fig. 4b). Osmoregulation should affect both otariid and phocid seals in similar ways through their food uptake during diving in a hypersaline environment (Costa 2018). Indeed, a previous study found evidence for accelerated evolution in an ion-transporter regulating fluid homeostasis in pinnipeds (Yuan *et al.* 2021). However, for example the extent of fasting and lack of water uptake while defending their harems in males is dependent on the mating system, which varies markedly among pinniped species (Berta 2018; Bowen 2018). Otariids and elephant seals are highly polygynous, with bulls fasting to defend their territories or harems, while the majority of phocids are less polygynous and the males do not fast for extended periods. This behavioral variability might drive the observed differentiation in osmoregulation-related BUSCOs.

Finally, one GO term mirrors the findings of the sliding window-based analysis. The GO term with the most significant enrichment based on the $F_{ST}$ cutoff (GO:0070098, F01) is linked to the chemokine-mediated signaling pathway. While chemokine-mediated signaling is generally involved in many biological processes, in hooded seals (*Cystophora cristata*), the upregulation of chemokines was observed in response to hypoxia and was linked to the formation of ROS after the reoxygenation of brain tissue (Hoff *et al.* 2017). Therefore, both the elevated $F_{ST}$ values around the GSTs (f1, Supplementary Fig. S6a), and the enrichment of chemokine-mediated signaling-related BUSCOs could be interpreted as an indication of differential adaptations to oxidative stress caused by different exposures to apnea-induced hypoxia in the Otariidae and the Phocidae.

### Conclusions

The field of population genomics is progressing at a stunning pace and many approaches that were only available to model systems are now becoming accessible for the study of wild populations. However, many of these approaches are dependent on the availability of high-quality reference genomes. In cetacean research, this realization has sparked a concerted genome assembly effort, which aims to provide reference genomes for many whale species that adhere to the quality standards of the vertebrate genome project (Morin *et al.* 2020; Rhie *et al.* 2021). Here, we combine long-read sequencing, haplotype-aware HiRise assembly and Hi-C-based mega-scaffolding to create a greatly improved chromosome-level Antarctic fur seal reference genome (arcGaz4_h1). This reference genome should serve as a valuable resource for population genomic studies of Antarctic fur seals specifically, and pinnipeds more generally. By building resources for pinniped genomics, we hope to foster the potential for broad comparative analyses in the field of marine mammal research, particularly, by complementing parallel developments in cetacean genomics. Our exploratory investigation into the phylogenetic context of the Antarctic fur seal genome highlights how the availability of high-quality genome assemblies can enable research beyond the confinements of particular species. We believe that our findings can serve as a starting point for more in-depth evolutionary studies and are thus looking forward to exciting times in the field of pinniped population genomics.

## Supplementary Material

jkae179_Supplementary_Data

## Data Availability

The genome assemblies arcGaz4_h1 and arcGaz4_h2 are deposited at NCBI under the accession numbers PRJNA1099197 and PRJNA1099198, while the raw sequencing data underlying the assemblies is deposited under PRJNA1134077. The initial *de novo* genome assemblies, as well as the annotation for arcGaz4_h1, the multi-species whole-genome alignment, conservation scores, $F_{ST}$ and GO term enrichment results are deposited at dryad, in the repository DOI: 10.5061/dryad.g1jwstqzn. The code for the analyses presented in this study is deposited at zenodo, in the repository DOI: 10.5281/zenodo.10979149. Supplemental material available at G3 online.
