# Supplementary material for: Refinement of the Antarctic fur seal (Arctocephalus gazella) reference genome increases continuity and completeness
Source: G3 (Bethesda). 2024 Jul 31;14(11):jkae179. doi: 10.1093/g3journal/jkae179 (PMC11540311; doi:10.1093/g3journal/jkae179)
Supplement: jkae179_Supplementary_Data [file jkae179_supplementary_data.pdf]

Supplemental Material for: Refinement of the Antarctic fur seal (*Arctocephalus gazella*) reference genome increases continuity and completeness

Kosmas Hench [*,1,2,3], David L.J. Vendrami [1,2], Jaume Forcada [4] and Joseph I. Hoffman [1,2,4,5,6]

[1]Department of Evolutionary Population Genetics, Faculty of Biology, Bielefeld University, 33501 Bielefeld, Germany
[2]Department of Animal Behaviour, Bielefeld University, 33501 Bielefeld, Germany
[3]Museum für Naturkunde - Leibniz Institute for Evolution and Biodiversity Science, Invalidenstr. 43, 10115 Berlin.
[4]British Antarctic Survey, UKRI-NERC, High Cross, Madingley Road, Cambridge CB3 0ET, UK.
[5]Center for Biotechnology (CeBiTec), Faculty of Biology, Bielefeld University, 33615 Bielefeld, Germany
[6]Joint Institute for Individualisation in a Changing Environment (JICE), Bielefeld University and University of Münster, 33501 Bielefeld, Germany

*Corresponding author: k.hench@posteo.de

Supplementary Information

Supplementary Figures

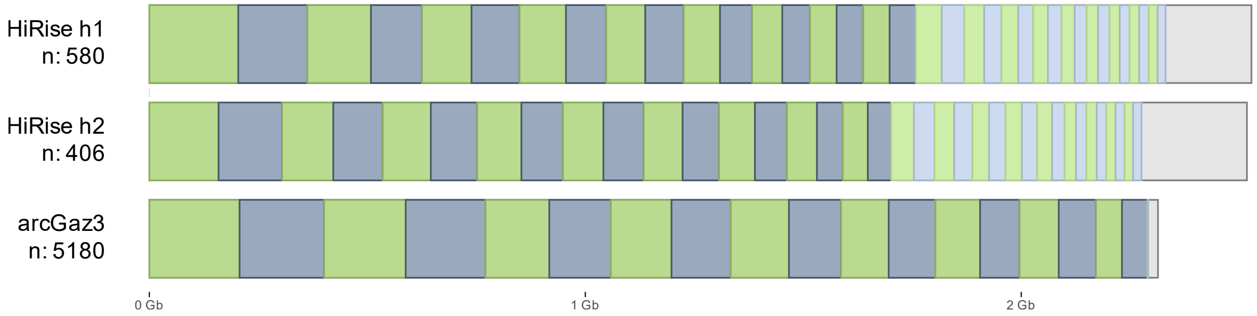

Figure S1 Comparison of the initial HiRise haplotype assemblies and the previous *A. gazella* reference genome. The previous reference genome (arcGaz3) is shown in the bottom row, while above the two new haplotype assemblies prior to the synteny based anchoring step are displayed. The scaffolds are arranged by decreasing length, with blocks of alternating colors representing the largest individual scaffolds. Darker shades indicate the 18 largest scaffolds, lighter shades indicate up to the 36th largest scaffold. The trailing gray blocks indicate the cumulative length of the remaining smaller scaffolds, so that the overall assembly length is given by the total length of entire row. Genome size in giga bases (Gb) is indicated below.

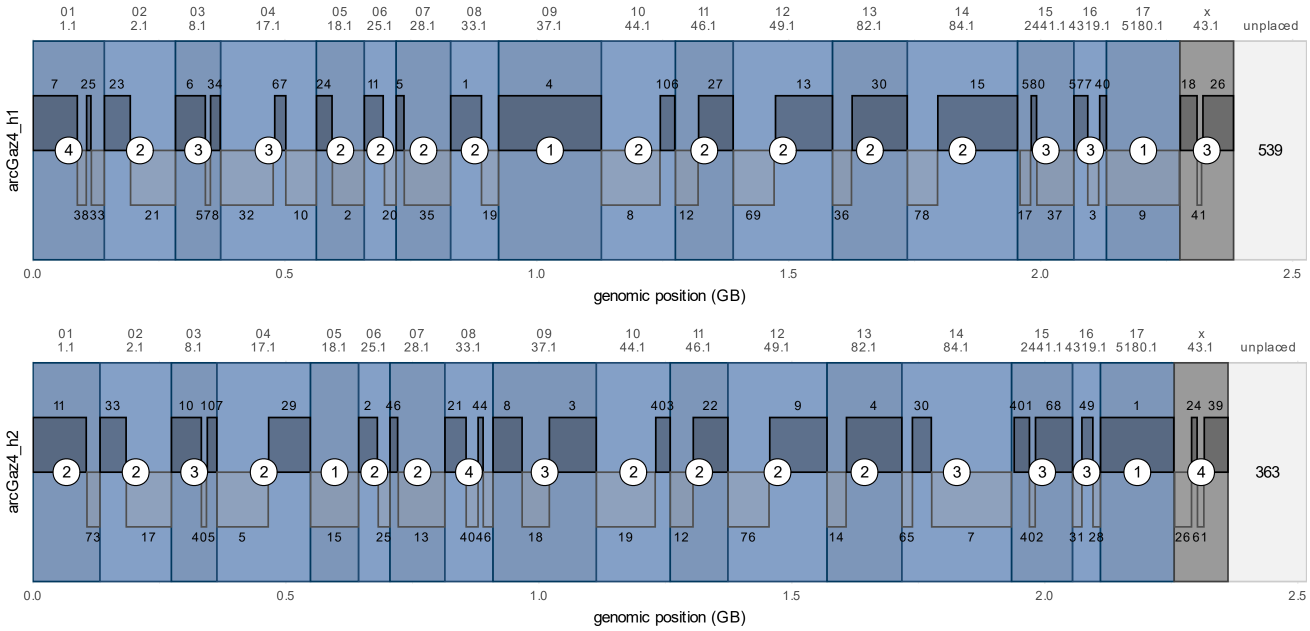

Figure S2 Synteny based anchoring of the HiRise haplotype assemblies. The top row represents haplotype 1 of the anchored HiRise assemblies, the bottom row represents haplotype 2. Within each row, the inner alternating small bars indicate the original scaffolds from the initial HiRise assembly and the background bars indicate the resulting anchored "mega-scaffolds" in arcGaz4_h1 (top) and arcGaz4_h2 (bottom). The number of in the circle displays the number of anchored scaffolds within each mega-scaffold and the numbers above and below the bars are the respective scaffold IDs. Above each mega-scaffold, the new ID is given above the ID of the underlying scaffold from the arcGaz3 assembly that was used for the anchoring ('01'–'x' refers to scaffolds mscaf_a1_01–mscaf_a1_x and '1.1'–'43.1' to CAAAJK-010000001.1–CAAAJK-010000043.1). The trailing light-gray bars indicate the extent of unplaced scaffolds that were carried over untouched from the initial HiRise assemblies.

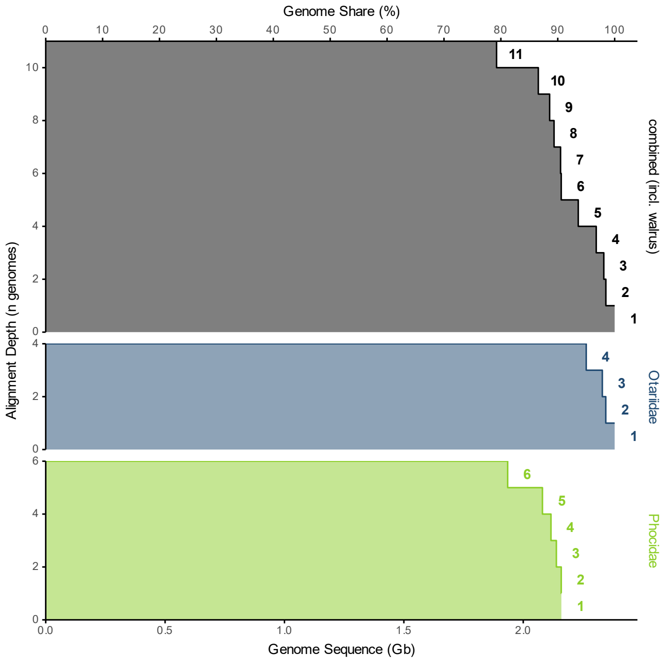

Figure S3 Alignment depth. Coverage of the `cactus` alignment on the Antarctic fur seal reference genome. The black bars in the upper panel indicate the overall alignment depth, the blue bars the depth of otariid genomes (with a maximum of four) and the green bars the alignment depth of phocid genomes (with a maximum of six). Note, that the individual tracks represent the cumulative alignment coverage over all genomes, and not individual genomes. In total eleven genomes were aligned (otariid and phocid genomes as well as the walrus), so 11 is also the maximal possible coverage. In both the complete and the otariid set, a coverage of one indicates that no genomes are aligned at those positions of the Antarctic fur seal genome and the alignment contains only the sequence of the reference genome.

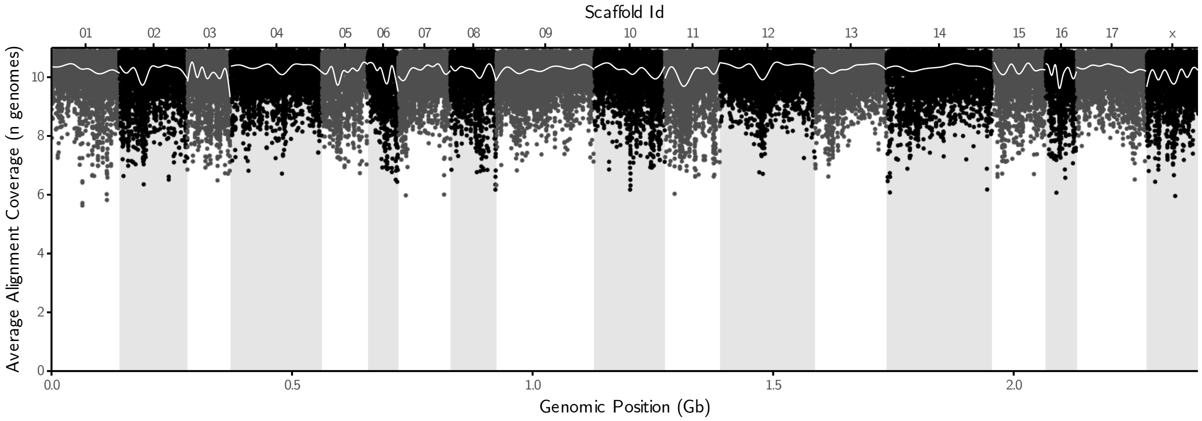

Figure S4 Alignment depth along the genome. Average depth of the cactus alignment within 50 kb windows with 25 kb increments along the Antarctic fur seal reference genome. The white line indicates a gam-smoothing of the windowed averages. Alternating white and gray backgrounds indicate the 18 major scaffolds with the scaffold labels on top ('01'–'x' refers to scaffolds mscaf_a1_01–mscaf_a1_x).

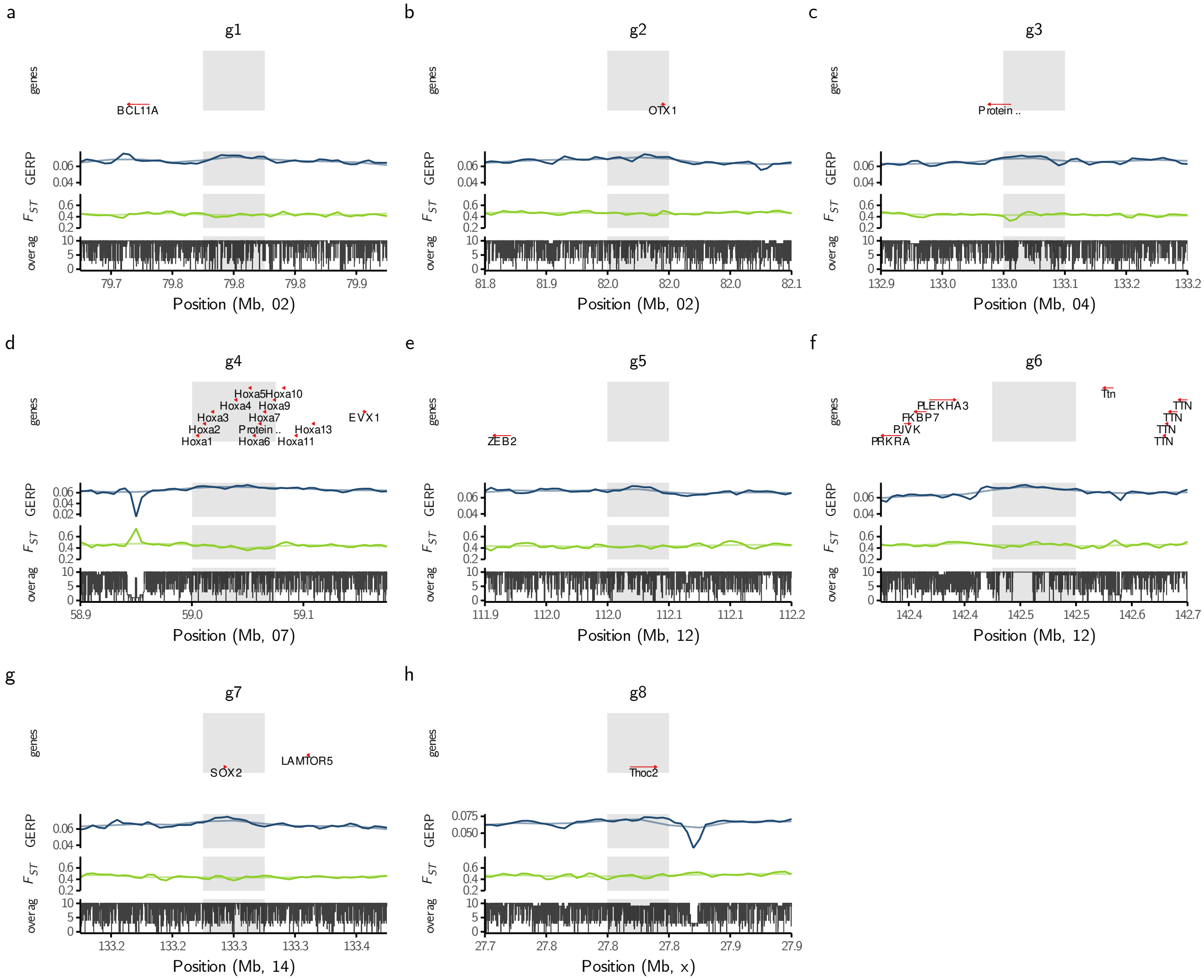

Figure S5 Zoom on GERP score outlier regions. The eight panels **a**–**h** are zooms on the peaks in the average GERP RS score identified in **Figure ??** b (g1–g8). The central gray background shading indicates the extent of the outlier regions based on the 50 kb windows exceeding the top 0.01 %ile of average GERP RS scores throughout the genome. The top track of each panel identifies gene models within the outlier regions according to the genome annotation. The 50 kb windowed averages for both GERP and F_{ST} values are depicted using the faint blue (GERP across all pinnipeds) and green (F_{ST} between Otariidae and Phocidae) lines. They are overlayed with a darker and more detailed 10 kb windowed averages. The bottom track of each panel indicates the alignment depth (n genomes) within the region.

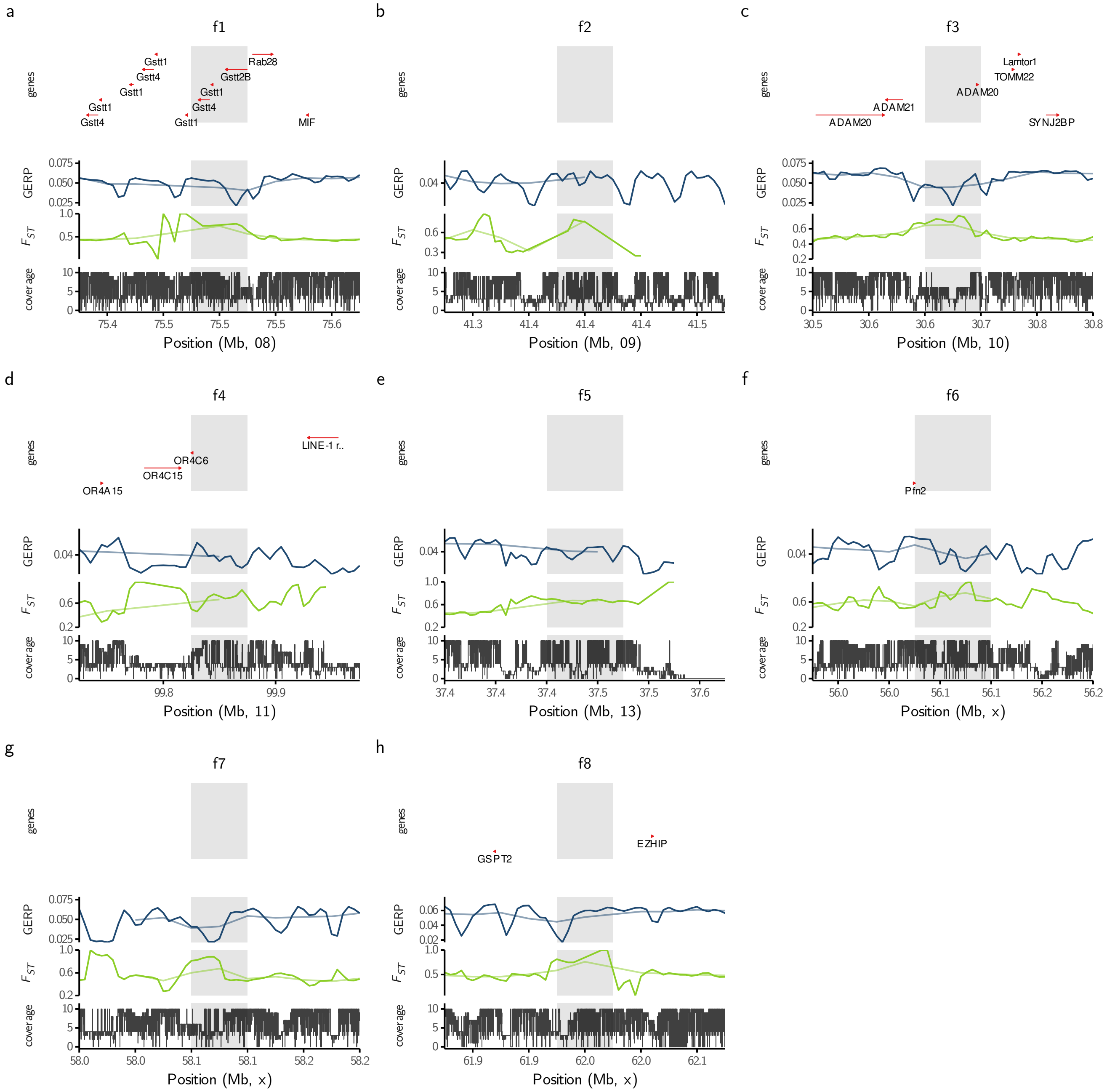

Figure S6 Zoom on F_{ST} outlier regions. The eight panels **a–h** are zooms on the peaks in the average F_{ST} value identified in **Figure ??** d (f1–f8). The central gray background shading indicates the extent of the outlier regions based on the 50 kb windows exceeding the top 0.01 %ile of average F_{ST} values throughout the genome. The top track of each panel identifies gene models within the outlier regions according to the genome annotation. The 50 kb windowed averages for both GERP and F_{ST} values are depicted using the faint blue (GERP across all pinnipeds) and green (F_{ST} between Otariidae and Phocidae) lines. They are overlayed with a darker and more detailed 10 kb windowed averages. The bottom track of each panel indicates the alignment depth (n genomes) within the region. Note that drops in alignment coverage can cause gaps in both F_{ST} and GERP summaries, particularly if coverage is restricted to one of the two pinniped families.

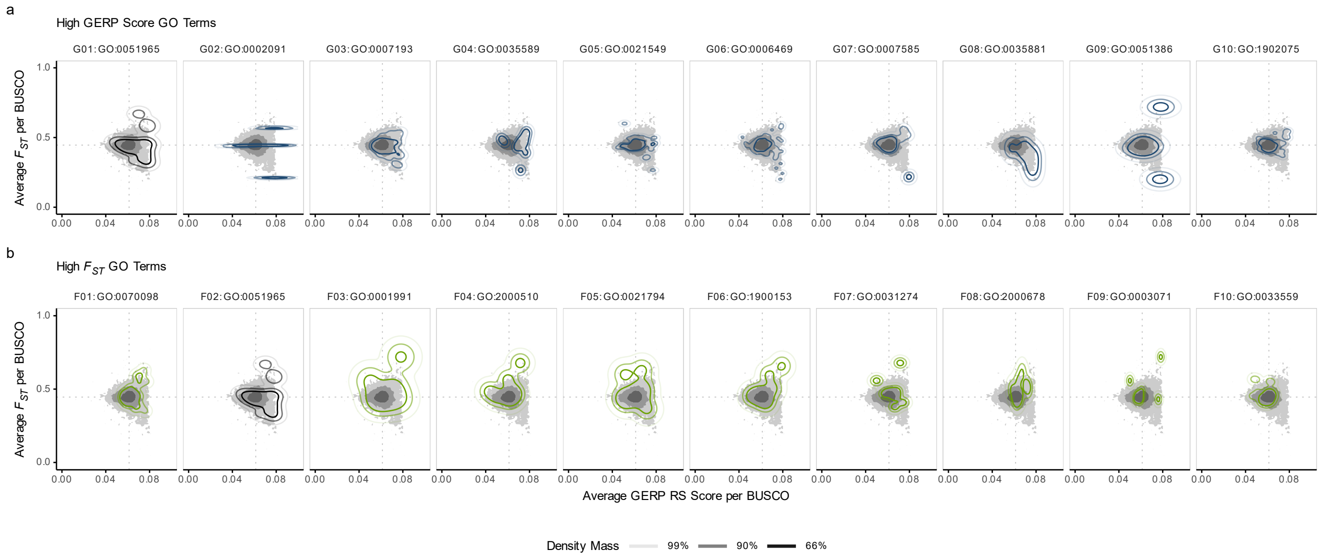

Figure S7 Bivariate density distribution of average Genomic Evolutionary Rate Profiling (GERP) scores and F_{ST} among BUSCOs. The bivariate densities summarize the average conservation scores (x) and F_{ST} values (y) among BUSCOs. The dotted lines indicate the median GERP and F_{ST} values among the full BUSCO set. **a** Density distributions for BUSCOs linked to the top ten GO terms of the GERP based enrichment analysis. The blue lines represent density isolines of the GO term specific BUSCO subset. **b** Density distributions for BUSCOs linked to the top ten GO terms of the F_{ST} based enrichment analysis. The green lines represent density isolines of the GO term specific BUSCO subset. Note, that GO term GO:0070098 is indicated in black, since it is included in both top ten GO term sets. For context, the density of all BUSCOs is given in the background of all panels.

Supplementary Tables

Table S1 Pinniped genomes included in the cactus alignment. Within the hal file, the tag in the column “Label” is used to identify the genomes.

Species	Label	NCBI accession	Family	Species	Label	NCBI accession	Family
Arctocephalus gazella	arcgaz	arcGaz4_h1, *in process*	Otariidae	*Halichoerus grypus*	halgry	GCF_012393455.1	Phocidae
Callorhinus ursinus	calurs	GCF_003265705.1	Otariidae	*Leptonychotes weddellii*	lepwed	GCF_000349705.1	Phocidae
Eumetopias jubatus	eumjub	GCF_004028035.1	Otariidae	*Mirounga angustirostris*	mirang	GCF_021288785.2	Phocidae
Zalophus californianus	zalcal	GCF_009762305.2	Otariidae	*Mirounga leonina*	mirleo	GCF_011800145.1	Phocidae
Odobenus rosmarus	odoros	GCF_000321225.1	Odobenidae	*Neomonachus schauinslandi*	neosch	GCF_002201575.2	Phocidae
				Phoca vitulina	phovit	GCF_004348235.1	Phocidae

Table S2 Description of the top ten GO terms identified by the enrichment analysis based on the most conserved BUSCOs (top 1%). The "Term" and "Description" columns originate from QuickGO (accessed 2023-12-13, **?**). Note that the "Rank" columns include rank ties and that GO term GO:0051965 is also included in **Table S3** as F02.

Label	GO Term	GERP		F_{ST}		Term	Description
		Rank	*p* value	Rank	*p* value		
G01	GO:0051965	1	0.0000	2	0.0017	positive regulation of synapse assembly	Any process that activates, maintains or increases the frequency, rate or extent of synapse assembly, the aggregation, arrangement and bonding together of a set of components to form a synapse.
G02	GO:0002091	2	0.0000	46	0.0455	negative regulation of receptor internalization	Any process that stops, prevents, or reduces the frequency, rate or extent of receptor internalization.
G03	GO:0007193	3	0.0001	1471	1.0000	adenylate cyclase-inhibiting G protein-coupled receptor signaling pathway	A G protein-coupled receptor signaling pathway in which the signal is transmitted via the inhibition of adenylyl cyclase activity and a subsequent decrease in the intracellular concentration of cyclic AMP (cAMP).
G04	GO:0035589	4	0.0002	1471	1.0000	G protein-coupled purinergic nucleotide receptor signaling pathway	A G protein-coupled receptor signaling pathway initiated by an extracellular purine nucleotide binding to its receptor, and ending with the regulation of a downstream cellular process.
G05	GO:0021549	5	0.0002	784	0.3963	cerebellum development	The process whose specific outcome is the progression of the cerebellum over time, from its formation to the mature structure. The cerebellum is the portion of the brain in the back of the head between the cerebrum and the pons. In mice, the cerebellum controls balance for walking and standing, modulates the force and range of movement and is involved in the learning of motor skills.
G06	GO:0006469	6	0.0002	210	0.1001	negative regulation of protein kinase activity	Any process that stops, prevents, or reduces the frequency, rate or extent of protein kinase activity.
G07	GO:0007585	7	0.0013	406	0.1779	respiratory gaseous exchange by respiratory system	The process of gaseous exchange between an organism and its environment. In plants, microorganisms, and many small animals, air or water makes direct contact with the organism’s cells or tissue fluids, and the processes of diffusion supply the organism with dioxygen (O_2) and remove carbon dioxide (CO_2). In larger animals the efficiency of gaseous exchange is improved by specialized respiratory organs, such as lungs and gills, which are ventilated by breathing mechanisms.
G08	GO:0035881	8	0.0016	1471	1.0000	amacrine cell differentiation	The process in which a relatively unspecialized cell acquires specialized features of an amacrine cell, an interneuron generated in the inner nuclear layer (INL) of the vertebrate retina. Amacrine cells integrate, modulate, and interpose a temporal domain in the visual message presented to the retinal ganglion cells, with which they synapse in the inner plexiform layer. Amacrine cells lack large axons.
G09	GO:0051386	8	0.0016	79	0.0544	regulation of neurotrophin TRK receptor signaling pathway	Any process that modulates the frequency, rate or extent of the neurotrophin TRK receptor signaling pathway.
G10	GO:1902075	10	0.0020	1471	1.0000	cellular response to salt	Any process that results in a change in state or activity of a cell (in terms of movement, secretion, enzyme production, gene expression, etc.) as a result of a salt stimulus.

Table S3 Description of the top ten GO terms identified by the enrichment analysis based on the most differentiated BUSCOs (top 1%). The "Term" and "Description" columns originate from QuickGO (accessed 2023-12-13, **?**). Note that the "Rank" columns include rank ties and that GO term GO:0051965 is also included in **Table S2** as G01.

Label	GO Term	GERP		F_{ST}		Term	Description
		Rank	*p* value	Rank	*p* value		
F01	GO:0070098	141	0.0538	1	0.0000	chemokine-mediated signaling pathway	The series of molecular signals initiated by a chemokine binding to its receptor on the surface of a target cell, and ending with the regulation of a downstream cellular process, e.g. transcription.
F02	GO:0051965	1	0.0000	2	0.0017	positive regulation of synapse assembly	Any process that activates, maintains or increases the frequency, rate or extent of synapse assembly, the aggregation, arrangement and bonding together of a set of components to form a synapse.
F03	GO:0001991	11	0.0022	3	0.0017	regulation of systemic arterial blood pressure by circulatory renin-angiotensin	The process in which angiotensinogen metabolites in the bloodstream modulate the force with which blood passes through the circulatory system. The process begins when renin is released and cleaves angiotensinogen.
F04	GO:2000510	1603	1.0000	3	0.0017	positive regulation of dendritic cell chemotaxis	Any process that activates or increases the frequency, rate or extent of dendritic cell chemotaxis.
F05	GO:0021794	1603	1.0000	5	0.0023	thalamus development	The process in which the thalamus changes over time, from its initial formation to its mature state.
F06	GO:1900153	223	0.0804	5	0.0023	positive regulation of nuclear-transcribed mRNA catabolic process, deadenylation-dependent decay	Any process that activates or increases the frequency, rate or extent of nuclear-transcribed mRNA catabolic process, deadenylation-dependent decay.
F07	GO:0031274	1603	1.0000	7	0.0029	positive regulation of pseudopodium assembly	Any process that activates or increases the frequency, rate or extent of the assembly of pseudopodia.
F08	GO:2000678	1603	1.0000	7	0.0029	negative regulation of transcription regulatory region DNA binding	Any process that stops, prevents or reduces the frequency, rate or extent of transcription regulatory region DNA binding.
F09	GO:0003071	23	0.0046	9	0.0036	renal system process involved in regulation of systemic arterial blood pressure	Renal process that modulates the force with which blood travels through the circulatory system. The process is controlled by a balance of processes that increase pressure and decrease pressure.
F10	GO:0033559	1603	1.0000	10	0.0048	unsaturated fatty acid metabolic process	The chemical reactions and pathways involving an unsaturated fatty acid, any fatty acid containing one or more double bonds between carbon atoms.
